# Study protocol of a randomized controlled trial comparing Mindfulness-Based Stress Reduction with treatment as usual in reducing psychological distress in patients with lung cancer and their partners: the MILON study

**DOI:** 10.1186/1471-2407-14-3

**Published:** 2014-01-03

**Authors:** Melanie PJ Schellekens, Desiree GM van den Hurk, Judith B Prins, Johan Molema, A Rogier T Donders, Willem H Woertman, Miep A van der Drift, Anne EM Speckens

**Affiliations:** 1 Department of Psychiatry, Radboud University Nijmegen Medical Centre, Nijmegen, The Netherlands; 2 Department of Pulmonary Diseases, Radboud University Nijmegen Medical Centre, Nijmegen, The Netherlands; 3 Department of Medical Psychology, Radboud University Nijmegen Medical Centre, Nijmegen, The Netherlands; 4 Department of Epidemiology, Biostatistics, and Health Technology Assessment, Radboud University Nijmegen Medical Centre, Nijmegen, The Netherlands

**Keywords:** Mindfulness-based stress reduction, Lung cancer patients, Partners, Psychological distress, Randomized controlled trial

## Abstract

**Background:**

Lung cancer is the leading cause of cancer death worldwide and characterized by a poor prognosis. It has a major impact on the psychological wellbeing of patients and their partners. Recently, it has been shown that Mindfulness-Based Stress Reduction (MBSR) is effective in reducing anxiety and depressive symptoms in cancer patients. The generalization of these results is limited since most participants were female patients with breast cancer. Moreover, only one study examined the effectiveness of MBSR in partners of cancer patients. Therefore, in the present trial we study the effectiveness of MBSR versus treatment as usual (TAU) in patients with lung cancer and their partners.

**Methods/Design:**

A parallel group, randomized controlled trial is conducted to compare MBSR with TAU. Lung cancer patients who have received or are still under treatment, and their partners are recruited. Assessments will take place at baseline, post intervention and at three-month follow-up. The primary outcome is psychological distress (i.e. anxiety and depressive symptoms). Secondary outcomes are quality of life (only for patients), caregiver appraisal (only for partners), relationship quality and spirituality. In addition, cost-effectiveness ratio (only in patients) and several process variables are assessed.

**Discussion:**

This trial will provide information about the clinical and cost-effectiveness of MBSR compared to TAU in patients with lung cancer and their partners.

**Trial registration:**

ClinicalTrials.gov NCT01494883.

## Background

With an estimated 1.4 million deaths per year, lung cancer is the leading cause of death by cancer worldwide. Even with the best available treatment, five-year survival is merely 16% and about 60 to 70% of patients die within the first year after diagnosis [1]. This poor prognosis is often caused by a late diagnosis as the presentation usually occurs when the lung cancer is advanced. Patients may develop burdensome symptoms like pain, dyspnoea, fatigue and cough and they may undergo radical treatment, including surgery, chemo- and radiotherapy. Not surprisingly, lung cancer has a major impact on the psychological wellbeing of patients and their family. Akechi and colleagues [2] showed that 19% of patients with advanced lung cancer meets the criteria of psychiatric disorders, especially depressive and adjustment disorders. Of patients who had been successfully treated for lung cancer 15% met the criteria for a minor or major depressive disorder [3]. The prevalence rate of depressive and anxiety symptoms among lung cancer patients ranges from 20 to 47% [4-7]. Compared to patients with other cancer diagnoses, lung cancer patients report the highest rates of distress (43 to 58%) [8,9] resulting in a lower quality of life [10].

Family, friends and especially partners of patients with lung cancer also have to deal with its psychological impact [11-14]. Partners not only provide emotional and practical support, they also have to cope with their own concerns, including the uncertainty regarding the course of the illness and the fear of losing their partner [15]. More than 50% of partners of lung cancer patients report negative emotional effects of caregiving [16]. Around 40% of partners of patients with advanced lung cancer report high levels of distress [17]. The relationship between patient and partner can also be affected by the cancer. It has been shown that some partners report a lower quality of their relationship after the diagnosis of lung cancer [18].

Though numerous studies examined the psychological distress of lung cancer patients and their partners [2-22], not much research is done on how to alleviate distress in these groups [23]. In addition, the available studies on managing the psychosocial care needs of cancer patients and their families have focused on care at the very end of life (e.g. [24-26]). Recently, studies have demonstrated that palliative care initiated early in treatment improves the quality of life and depressive symptoms of lung cancer patients [10,27]. This stresses the importance of integrating psychosocial care for lung cancer patients and their partners early in the treatment, rather than instigating it once life-prolonging therapies fail.

In the past ten years MBSR has become a promising psychosocial intervention for cancer patients. Mindfulness is defined as intentionally paying attention to moment-by-moment experiences in a non-judgmental way [28]. MBSR is an 8-week group-based training consisting of meditation practices, such as the bodyscan, gentle yoga, sitting and walking meditation. By repeatedly bringing attention back to the current experience, participants gradually learn to disengage from dysfunctional thoughts and directly experience the emotions and bodily sensations of the present moment. MBSR aims to provide participants with the ability to step back from ruminating about the past or worrying about the future and simply allow experiences to unfold [28,29]. A recent meta-analysis [30] of 13 nonrandomized studies and 9 randomized controlled trials (RCT) concluded there is positive evidence for the use of mindfulness-based interventions in reducing psychological distress in cancer patients. Among the RCT’s, a reduction in symptom severity was found for both anxiety and depression corresponding to moderate pooled controlled effect sizes (Hedges’s *g =* 0.37 and Hedges’s *g* = 0.44, respectively) [30]. Though mindfulness-based interventions seem to be effective, the authors note that across studies the majority of participants were women (85%) and diagnosed with breast cancer (77%). Compared to breast cancer patients, patients with lung cancer are more often male, older and have a poorer prognosis. Furthermore, of these 22 studies only one study included the partners of the patients showing that partners also benefit from the MBSR training [31]. This is quite surprising since partners of cancer patients also report high levels of distress [32].

### Aims

The aim of the Mindfulness for Lung Oncology Nijmegen (MILON) study is to examine the effectiveness of MBSR compared to TAU in reducing psychological distress in patients with lung cancer and their partners. We hypothesize that patients in the MBSR group will report a lower level of psychological distress (i.e. anxiety and depressive symptoms), higher levels of quality of life, quality of relationship and spirituality than those in the TAU group. Medical and societal costs will be lower in the MBSR versus TAU group. We expect partners in the MBSR group to report a lower level of psychological distress and higher levels of caregiver appraisal, relationship quality and spirituality than their counterparts in the TAU group. With regard to the working mechanisms of the MBSR programme, we will examine changes in mindfulness skills, self-compassion, rumination, intrusion, avoidance and adherence to MBSR.

## Methods/Design

### Study design

The design of the ‘MILON’ study is a parallel group randomized controlled trial with an embedded process study. Participants are randomized between MBSR and TAU. The study protocol has been approved by our ethical review board (CMO Arnhem-Nijmegen) and registered under number 2011–519.

### Participants and procedure

Patients and partners are recruited at the outpatient clinic of the Department of Pulmonary Diseases, Radboud University Nijmegen Medical Centre (RUNMC) by a nurse practitioner and the attending physician. Patients and partners are invited to participate together but both are welcome to participate on their own if they do not have a partner or their partner is not willing to participate. Patients and/or partners who are interested are provided with an information leaflet. If they are willing to participate, they are invited for a research interview, in which in- and exclusion criteria are assessed and informed consent is taken.

At other participating hospitals (Department of Pulmonary Diseases, Canisius-Wilhelmina Hospital, Nijmegen; Department of Pulmonary Medicine, Rijnstate, Arnhem; Department of Oncology, Elkerliek Hospital, Helmond; Department of Pulmonary Medicine, Jeroen Bosch Hospital; Department of Pulmonary Diseases, Maas hospital Pantein, Boxmeer) patients and their partners will be sent a letter with the invitation to participate in the study. One week later the researcher calls the patients to answer possible questions and asks whether the patient and partner are interested in participation. If so, they are invited for a research interview at the RUNMC.

#### Eligibility

We include patients and/or partners of patients, who are (a) diagnosed with cytologically or histologically proven non-small cell lung cancer or small cell lung cancer and (b) have received or are still under treatment. Exclusion criteria for both patient and partner include: (a) being under 18 years of age, (b) not being able to understand or use the Dutch language, (c) former participation in MBSR or Mindfulness-Based Cognitive Therapy (MBCT), (d) current and regular treatment by psychologist or psychiatrist, (e) current participation in other psychosocial programme and (f) physical or cognitive (<26 on the Mini-Mental State Examination (MMSE)) impairments hampering participation in MBSR training or completion of questionnaires.

#### Baseline

Patients and partners are interviewed to obtain demographics and clinical characteristics, after which they are screened for cognitive impairments with the MMSE [33]. After that, baseline questionnaires, including the Distress Thermometer (DT) [34,35], are administered, followed by randomization. Table 1 shows the assessment instruments and time points at which the questionnaires are administered to patients and partners.

**Table 1:** Measurements and corresponding time points for patient and partner

**Measure**	**Target**	**T0**		**T1**		**T2**	
		**pt**	**pr**	**pt**	**pr**	**pt**	**pr**
MMSE	Cognitive impairments	x	x				
DT	General distress	x	x				
HADS	Psychological distress	x	x	x	x	x	x
QLQ-C30	Quality of life	x		x		x	
QLQ-LC13	Quality of life	x		x		x	
SIP	Impact of sickness	x		x		x	
SPPIC	Caregiver burden		x		x		x
CRA-SE	Caregiver self-esteem		x		x		x
IMS-S	Relationship satisfaction	x	x	x	x	x	x
MIS	Communication about cancer	x	x	x	x	x	x
SAIL	Spirituality	x	x	x	x	x	x
FFMQ	Mindfulness skills	x	x	x	x	x	x
SCS	Self-compassion	x	x	x	x	x	x
RRS-EXT	Rumination	x	x	x	x	x	x
IES	Psychological stress reaction	x	x	x	x	x	x
Diary	Health care use, work absence	Monthly during study period for pt					
Calendar	Mindfulness adherence	Monthly during study period for pt and pr					

*Note.* T0 = Baseline measurement; T1 = Post-intervention measurement; T2= 3-month follow-up measurement; pt = Patient; pr = Partner; MMSE = Mini Mental State Examination; DT = Distress Thermometer; HADS = Hospital Anxiety and Depression Scale; QLQ-C30 = Quality of Life – Cancer; QLQ-LC13 = Quality of Life – Lung Cancer; SIP = Sickness Impact Profile; SPPIC = Self-Perceived Pressure from Informal Care; CRA-SE = Caregiver Reaction Assessment – Care-Derived Self-Esteem; IMS-S = Investment Model Scale-Satisfaction; MIS = Mutuality and Interpersonal Sensitivity; SAIL = Spiritual Attitude and Involvement List; FFMQ = Five Facet Mindfulness Questionnaire; SCS = Self-Compassion Scale; RRS-EXT = Rumination Response Scale – Extended Version; IES = Impact of Event Scale.

#### Randomization

Randomization is stratified according to setting and minimized for (a) stage of disease (curative *versus* palliative), (b) baseline level of anxiety and depressive symptoms (anxiety or depression subscale score of Hospital Anxiety and Depression Scale (HADS) <8 *versus* ≥8), (c) treatment during MBSR (no treatment *versus* chemo- and/or radiotherapy) and (d) participation (patient alone *versus* partner alone *versus* patient and partner together). Randomization is computerized, using a randomization website, specifically designed for this study, on which the researcher can fill out the required data. The researcher communicates treatment allocation to the nurse practitioner, who informs the patient and/or partner.

#### Follow-up assessments

Follow-up assessments take place post intervention and at three-month follow-up. Participants who have access to the internet and have an email address receive the questionnaires online. If not, they receive the questionnaires on paper along with a reply envelope. In case of drop-out, the researcher tries to contact the participant by phone to complete a minimum set of outcome measures and to identify the main reason for drop-out.

### Intervention

The MBSR curriculum used is primarily based on the Mindfulness-Based Stress Reduction programme as developed by Kabat-Zinn [28] but contains some elements of the MBCT programme by Segal, Williams and Teasdale [29], like psycho-education on the interrelatedness of feelings and thoughts. Moreover, some modifications have been made to make the intervention more suitable for patients with lung cancer and their partners, such as psycho-education about grief [36]. In addition, a mindful communication exercise in which partners talk with each other about the cancer was added. The programme consists of 8 weekly 2.5-hour sessions, a silent day between session six and seven and home practice assignments of about 45 minutes, 6 days per week. Participants receive a set of CDs with guided mindfulness meditation exercises for home practice and a folder with information and home practice instructions for the forthcoming week. Table 2 shows the content of the MBSR programme per session. The MBSR courses are taught by mindfulness teachers with extensive training in MBSR. They all fulfil the advanced criteria of the Center for Mindfulness of the University of Massachusetts Medical School [37] and maintain a regular personal meditation practice. Teachers were trained, supervised and assessed to ensure their competency levels met the qualification criteria to instruct the MBSR classes. During the trial, teachers will receive weekly supervision and a number of sessions will be videotaped to evaluate competence and adherence with the Mindfulness-Based Interventions – Teaching Assessment Criteria [38].

**Table 2:** Content of MBSR programme per session

**Theme of session**	**Meditation exercise**	**Didactic teaching**	**Homework**
1. Automatic pilot	- Bodyscan	- Intention of participating	- Bodyscan
		- Raisin exercise	- Eating one meal mindfully
			- Attention for routine activity
2. Mindfulness of the breath	- Bodyscan	- Imagery exercise to demonstrate relationship between thoughtsand feelings	- Bodyscan
	- Sitting mediation with focus on breath		- Attention for breath
			- Awareness of pleasant events
			- Attention for routine activity
3. Observing limits	- Yoga while lying down	- Seeing exercise to demonstrate difference between observation and interpretation	- Bodyscan or yoga
	- 3-min breathing space		- Sitting meditation
			- Awareness of unpleasant events
			- 3-min breathing space
4. Opening up to distress	- Sitting mediation with focus on breath, body and sound	- Interrelatedness of feelings, thoughts and bodily sensations	- Bodyscan or yoga
			- Sitting meditation
	- 3-min breathing space	- Psychoeducation about grief	- Awareness of stress reactions
			- 3-min breathing space
5. Responding to distress	- Sitting mediation with focus on breath, body, sound, thoughts, difficulty	- Reacting versus responding	- Meditation by choice
		- Coping with grief	- Awareness of reaction in difficult situation
	- Walking meditation		- Awareness of communication difficulties
	- 3-min breathing space		- 3-min breathing space
6. Mindful communication	- Yoga in standing position	- Mindful communication exercise about effect of lung cancer with their own partner	- Sitting meditation or yoga
	- 3-min breathing space		- Awareness of communication
			- 3-min breathing space during stress
Silent day	- Varying meditation exercises		
	- Silent lunch and tea break		
7. Taking care of yourself	- Sitting meditation ending in choiceless awareness	- Exercise on taking care of yourself by examining how to improve balance in life	- Meditation without CD
	- Yoga or walking meditation		- Reflect on training
	- 3-min breathing space		
8. The rest of your life	- Bodyscan	- Reflection on training	- Further sources of information
	- Short sitting meditation	- Maintaining practice	

### Outcome measures

#### Primary outcome measure

##### Psychological distress

The primary outcome measure is the total score on the HADS [39-41], which is developed to measure psychological distress in somatic patient populations. It consists of a 7-item anxiety (HADS-A) and 7-item depression (HADS-D) subscale. The HADS shows good psychometric properties in the general medical population, including oncology patients [42]. Internal consistency as measured with Cronbach’s α varied from .84 to .90 [40,42].Test-retest reliability was good as Pearson’s *r* > .80 were obtained [40,43]. Though the cut-off scores of the HADS vary among populations [44], in lung cancer patients they have found to be <8 *versus* ≥8 on the HADS-A or HADS-D [45]. The HADS has been shown to be highly correlated with the Beck Depression Inventory [42]. It has previously been used in intervention studies of mindfulness and shown to be sensitive to change (e.g. [46]).

#### Secondary outcome measures

##### Quality of life (only for patients)

The European Organisation for Research and Treatment of Cancer (EORTC) Core Quality of Life Questionnaire (QLQ-C30) [47] is included, along with the supplemental Lung Cancer questionnaire module (QLQ-LC13) [48]. The QLQ-C30 is designed to use in clinical trials on physical treatments for cancer patients. It incorporates five functional scales (physical, role, cognitive, emotional, social), three symptom scales (fatigue, pain, nausea and vomiting), a global health and quality of life scale and an array of single-item symptom measures. After revisions in the role functioning, global health and physical functioning scale, internal consistency of the subscales varied between .65 and .94, except for the cognitive functioning scale with α varying from .56 to .63 [47,49,50]. Test-retest reliability varied from .63 to .86 [51]. The lung cancer questionnaire module is designed to supplement the core questionnaire and comprises specific symptoms associated with lung cancer (coughing, haemoptysis, dyspnoea, pain) and side-effects from conventional chemo- and radiotherapy (hair loss, neuropathy, sore mouth, dysphagia). While the multi-item dyspnoea scale showed high internal consistency, the pain subscale did not. When combined with the dyspnoea and pain items of the core questionnaire, both the dyspnoea (α = .86) and pain (α = .71) subscale showed high internal consistency. Since the QLQ-C30 and QLQ-LC13 are mainly focused on physical symptoms, we added the items Social Interaction and Alertness Behavior of the Sickness Impact Profile (SIP) [52]. Internal consistency was .94 and test-retest reliability was .92. The SIP correlated with self-assessed sickness and dysfunction [52].

##### Caregiver appraisal (only for partners)

We use the 9-item Self-Perceived Pressure from Informal Care (SPPIC) [53] to assess the extent to which caregiving is experienced as burdensome. To also measure positive aspects of caregiving, the 9-item subscale Care-Derived Self-Esteem of the Caregiver Reaction Assessment (CRA-SE) [54] is included. Internal consistency of the SPPIC was .79 and of the CRA-SE was .73. The SPPIC and CRA-SE were unrelated to each other [55].

##### Relationship quality

To measure relationship satisfaction we included the 10-item Satisfaction subscale of the Investment Model Scale (IMS-S) [56]. The IMS-S starts with 5 items that measure concrete examplars of satisfaction, to enhance the comprehensibility of the global items, which are utilized to form the construct. Internal consistency varied from .79 to .95 and the IMS-S was related to the Dyadic Adjustment Scale. Also, the Mutual Interpersonal Sensitivity scale (MIS) [57] is included to measure communication between partners about the cancer. It contains 18 items and is divided into two scales: open communication and avoiding negative thoughts about the cancer.

##### Spirituality

is measured with the Spiritual Attitude and Involvement List (SAIL) [58] and consists of 26 items, divided into the subscales meaningfulness, trust, acceptance, caring for others, connectedness with nature, transcendent experiences, and spiritual activities. The internal consistency varied from .74 to .88 and test-retest reliability varied from .77 to .92. All subscales, except for connectedness with nature, were related with the Functional Assessment of Chronic Illness Therapy – Spiritual Well-Being Scale.

#### Costs (only for patients)

The cost-effectiveness evaluation is carried out from a societal perspective, considering direct as well as indirect health costs. Data on costs are collected prospectively using a diary in which participants register a) health care utilization: the type of care and its duration, and b) cancer-related absence from work. Unit cost estimates are derived from the national manual for cost prices in the health care sector [59]. Costs of reduced ability to work are estimated using the friction costs method, which results in a more realistic estimate than the human capital approach [60]. Treatment costs of MBSR are calculated using activity-based-costing methods, thus measuring actual resources (time of therapist, time of patients, facilities) used. All unit cost prices are adjusted to 2013 prices. Unit cost estimates are combined with resource utilization data to obtain a net cost per patient over the entire follow-up period.

#### Process measures

##### Mindfulness skills

are examined with the 39-item Five Facet Mindfulness Questionnaire (FFMQ) [61,62]. The FFMQ is based on an exploratory factor analysis of five mindfulness measures, which allowed items from different instruments to form factors, providing an empirical integration of these independent attempts to operationalize mindfulness. This led to the following five subscales: observing, describing, acting with awareness, non-judging of inner experience and non-reactivity to inner experience. Internal consistency varied from .72 to .93 among the different subscales. Most subscales were related to meditation experience, Psychological Well-Being scales and psychological symptoms, including the Brief Symptom Inventory [61]. FFMQ is sensitive to change in mindfulness-based interventions and is found to mediate the relationship between mindfulness practice and improvements in psychological symptoms (e.g. [63]).

##### Self-compassion

is assessed with the Self Compassion Scale (SCS) [64,65], which has 26 items and is divided into six subscales: self-kindness versus self-judgment, common humanity versus isolation, and mindfulness versus over-identification. Internal consistency of the different subscales varied from .75 to .81 and test-retest reliability varied from .80 to .93. SCS correlated moderately with self-esteem measures, including the Rosenberg Self-Esteem Scale. Furthermore, whereas the self-esteem measures correlated significantly with the Narcissistic Personality Inventory, the SCS was unrelated to narcissism [64]. SCS is sensitive to change through mindfulness-based interventions and is found to mediate MBCT’s treatment effects [66].

To measure **rumination** we administered the extended version of the Ruminative Response Scale (RRS-EXT) [67], Raes and Hermans: The revised version of the Dutch Ruminative Response Scale, unpublished instrument]. The RRS-EXT contains 26 items in which a more adaptive thinking style (i.e. reflection) is distinguished from a more maladaptive one (i.e. brooding). Internal consistency varied from .72 to .77 and test-retest reliability varied from .60 to .62 for the brooding and reflection subscales. The concept of rumination seems to be sensitive to change through mindfulness-based interventions and has been shown to mediate the effect of MBSR on depressive symptoms in oncology patients [68].

The **psychological stress reaction** is measured with the 15-item Impact of Event Scale (IES) [69,70], which assesses two categories of responses: intrusive experiences and avoidance of thoughts and images associated with the event. Internal consistency varied from .65 to .92 [71] and test-retest reliability varied from .79 to .87 among the subscales [69]. IES correlated with anxiety and depression subscales of the General Health Questionaire.

##### Adherence to MBSR

is assessed during the entire study period with a calendar on which participants in the MBSR condition fill out on a daily basis whether they adhere to the mindfulness exercises: either formal practice (e.g. meditation exercise like the bodyscan), informal practice (e.g. activity with awareness) or no exercise. Adherence to MBSR has been shown to mediate the effects of MBCT on depressive symptoms [72].

### Statistical analysis plan

#### Sample size

To determine the required sample size, first the sample size was calculated that would be needed for a simple *t*-test and subsequently it was corrected for clustering, repeated measurements and baseline. A two-sided *t*-test on the total HADS score [39,40] (i.e. our primary outcome measure, examining psychological distress (HADS-total), anxiety symptoms (HADS-A) and depressive symptoms (HADS-D)) would require 64 participants in each group to have 80% power to detect a medium-sized difference (effect size = 0.5) with alpha = 0.05. To correct for clustering, we multiplied this sample size of 64 with the design factor (1 + (*n* − 1) * ICC), where *n* denotes the cluster size and where ICC denotes the intra-cluster correlation. In our study, the treatment groups will consist of 14 people, of whom about 7 will be patients. With *n* = 7 and an estimated ICC = 0.01. [72], the correction factor equals 1.06. To correct for repeated measurements and the use of the baseline measurement as a covariate, we multiplied the required sample size by the design factor $\left(\left(1+\rho \right)/2-\rho_{0}^{2}\right),$ where *ρ* denotes the correlation between the post-treatment HADS measurements, and *ρ*_0_ denotes the correlation between the baseline HADS with the post-treatment HADS measurements. With *ρ* = 0.8 and *ρ* = 0.5 as conservative estimates, the second design factor equals 0.65. Consequently, after correction for clustering and covariates, we arrived at a required sample size of 0.65 * 1.06 * 64 = 44 patients per arm. So 88 patients with lung cancer would be required for the study. Based on our pilot study [van den Hurk, Schellekens, Molema, Speckens and van der Drift, in preparation], we expect a 20% drop-out rate. Therefore, we intend to include 110 patients and 110 partners.

#### Primary analyses

The samples of lung cancer patients and partners will be analyzed separately. Baseline characteristics of the population will be compared between MBSR and control group to ensure that key variables were evenly distributed by randomization. First, analyses will be based on the intention-to-treat approach. Next, we will perform per-protocol analyses with the treatment-adherent sample (i.e. in the MBSR condition participants have to attend at least four of the eight MBSR sessions [73] and in the TAU condition participants do not attend a mindfulness-based programme).

We will use linear mixed models to analyze all outcome variables (i.e. psychological distress, quality of life (only for patient), caregiver appraisal (only for partner), relationship quality and spirituality), with treatment as fixed factor, baseline measurement as covariate and a random intercept based on MBSR group. This procedure will use all observed data in our analyses. In addition, Cohen’s *d* effect size [74] will be reported based on the difference between the group means on baseline and follow-up scores, divided by the pooled standard deviation at baseline and follow-up.

#### Secondary analyses

##### Cost effectiveness

The quality of life measures (i.e. QLQ-C30; QLQ-LC13) will be used to calculate Quality of Adjusted Life Years (QALYs) for each individual. Costs and effects (in terms of QALYs) will be combined in the incremental cost-effectiveness ratio (ICER). The ICER expresses cost-effectiveness in terms of incremental costs per QALY gained. To estimate confidence intervals for the mean of the ICER a non-parametric bootstrapping method will be used, performing 1000 replications of the original data. In order to express the implications of the cost-effectiveness results more clearly, a cost-acceptability curve will be constructed. In case of dominance, a full cost analysis will be conducted to estimate the mean savings per patient per year.

##### Mediation analyses

To examine the possible underlying mechanisms of change in MBSR, mediation analyses will be conducted. Only the data of the treatment-adherent sample will be included in these analyses. By means of a multiple mediation model suggested by Preacher and Hayes [75], we will test the mediating effect of mindfulness skills, self-compassion, rumination and adherence to MBSR on psychological distress, quality of life (only in patients), caregiver appraisal (only in partners), relationship quality and spirituality.

## Discussion

In the last ten years MBSR has not only proven to be a feasible and acceptable intervention in cancer patients [76], but it also seems to be effective in reducing psychological distress [30]. However, the generalization of these results is limited because most participants were female patients with breast cancer. A large part of lung cancer patients already have advanced cancer at time of diagnosis and are confronted with a poor prognosis and low health status. Consequently, they more often report psychological distress than patients with other diagnoses of cancer [8,9]. Hence, it is not yet clear whether MBSR is a feasible, acceptable and effective intervention in patients with lung cancer. Moreover, little is known about the effectiveness of MBSR in partners of cancer patients [30], though they also often report psychological distress.

Our pilot study of 19 lung cancer patients and 16 partners participating in an MBSR course, provides preliminary evidence that MBSR is feasible and acceptable in this population (van den Hurk, Schellekens, Molema, Speckens and van der Drift, in preparation). The current trial will answer the question whether MBSR is effective in patients with lung cancer and their partners.

We started enrolment of participants in February 2012. At the moment, we think recruiting a sufficient number of patients and partners will be a challenge due to rapidly fluctuating health status and sudden changes in cancer treatment [77]. The main reasons for declining participation in patients is ‘being too ill’ or that it is ‘too much of a burden during chemo and/or radiotherapy’. Furthermore, no perceived need or motivation for the training is commonly mentioned. Among partners, participation is highly depending on whether the patient is willing to participate. Although partners can take part separately, partners who are interested do often not participate when the patients decline participation.

Considering the difficulty of studying lung cancer patients and their partners [77], our trial will offer valuable information on whether MBSR, as one of the few available psychosocial care programmes, contributes to the alleviation of their psychological distress.

## Abbreviations

MBSR: Mindfulness-based stress reduction; RCT: Randomized controlled trial; RUNMC: Radboud University Nijmegen Medical Centre; MBCT: Mindfulness-based cognitive therapy; MMSE: Mini mental state examination; DT: Distress thermometer; HADS: Hospital anxiety and depression scale; QLQ-C30: Quality of life – cancer; QLQ-LC13: Quality of life – lung cancer; SIP: Sickness impact profile; SPPIC: Self-perceived pressure from informal care; CRA-SE: Caregiver reaction assessment – care-derived self-esteem; IMS-S: Investment model scale-satisfaction; MIS: Mutuality and interpersonal sensitivity; SAIL: Spiritual attitude and involvement list; FFMQ: Five facet mindfulness questionnaire; SCS: Self-compassion scale; RRS-EXT: Rumination response scale – extended version; IES: Impact of event scale.

## Competing interests

The authors declare that they have no competing interests.

## Authors’ contributions

All authors contributed to the design of the study. AS, MD and JP are the principal investigators of the study. MS drafted the paper, which was modified and supplemented by all other authors. DH, MS and MD are involved in recruiting participants while MS and DH take care of the logistics of the study and data collection. RD contributed specifically to the statistical analysis plan and WW contributed specifically to the design of the cost-effectiveness evaluation. All authors read and approved the final manuscript.

## Pre-publication history

The pre-publication history for this paper can be accessed here:

http://www.biomedcentral.com/1471-2407/14/3/prepub
